# Bibliometric analysis of hepatocellular carcinoma and tyrosine kinase inhibitors

**DOI:** 10.1097/MD.0000000000042015

**Published:** 2025-05-16

**Authors:** Wurihan Wu, Hejun Mao, Jian Song, Fan Yang

**Affiliations:** a Department of Neurology Department, Affiliated Hospital of Inner Mongolia Medical University, Hohhot, China; b Department of Hepatobiliary Surgery, Affiliated Hospital of Inner Mongolia Medical University, Hohhot, China; c Emergency Intensive Care Unit, Inner Mongolia Autonomous Region People’s Hospital, Hohhot, China.

**Keywords:** bibliometric analysis, hepatocellular carcinoma, tyrosine kinase inhibitors

## Abstract

**Background:**

Hepatocellular carcinoma (HCC) is a common malignant tumor globally and in China, and its incidence and mortality rate are increasing year by year, and it faces many challenges and difficulties in treatment. Tyrosine kinase inhibitors (TKIs) have important roles in cell growth, proliferation, and differentiation, and have now become important drugs for cancer treatment. There are no bibliometric studies on liver cancer and TKIs to date.

**Methods:**

We retrieved 2848 records from the Web of Science™ Core Collection (WoSCC) database and analyzed them scientifically and metrically using CiteSpace and VOSviewer in terms of temporal and spatial distributions, author distributions, journal distributions, references, and keywords.

**Results:**

From January 1, 2004, to December 31, 2023, the WoSCC database documented 2848 publications related to tyrosinase inhibitors in HCC, comprising 2151 articles and 697 reviews. This literature involved 80 countries and regions, 3265 institutions, and 16,653 authors. Analysis shows a steady increase in publications annually since 2004, divided into 3 phases: 2004 to 2010 with fewer than 100 papers annually, suggesting minimal research attention; 2011 to 2019 with gradual growth, indicating increasing research interest; and a rapid surge post-2020, peaking in 2023, signaling heightened global interest in this field.

**Conclusion:**

Our bibliometric analysis on TKIs and HCC spans years, countries, institutions, authors, disciplines, and journals. Since 2004, this field has gained attention, with current research focusing on inflammatory and immune mechanisms, associated diseases, cytokines, and TKIs’ applications in liver cancer treatment, including combination therapies. These areas signify emerging research directions.

## 1. Introduction

Liver cancer is the 6th most common cancer in the world and has been a global challenge as one of the third most common tumors with a mortality rate after gastric and esophageal cancers. The World Health Organization has estimated that more than 1 million people will die of liver cancer in 2030 based on annual projections.^[1]^ Hepatocellular carcinoma (HCC) is the most common pathological type of liver cancer, accounting for 75% of all liver cancers, and its occurrence is associated with a variety of liver diseases such as viral hepatitis, alcoholic and nonalcoholic fatty liver disease. Tumor growth is dependent on direct contact or paracellular effects related to tumor cells, immune cells, and vascular endothelial cells in the tumor microenvironment.^[2]^ Traditional surgical resection, transcatheter arterial chemoembolization, percutaneous anhydrous ethanol injection, radiofrequency ablation therapy, and liver transplantation are the main treatment modalities for HCC,^[3]^ and surgery is feasible to achieve a complete cure for early-stage HCC. Early-stage liver cancer can be treated with surgery to achieve a complete cure, but 70% of liver cancer patients have already progressed to the middle or late stage at the time of consultation, losing the opportunity for surgery.

With the development of molecular biology and immunology, molecularly targeted drugs such as multireceptor tyrosine kinase inhibitors (TKI) have gradually become one of the main approaches for the treatment of intermediate and advanced HCC in recent years.^[4]^ Receptor tyrosine kinases (RTKs) are a group of transmembrane receptors characterized by their cytoplasmic region with intrinsic activity of tyrosine kinases (TKs). RTKs are expressed during prenatal and postnatal development of multicellular animals and play important roles in pathways such as cell survival, proliferation, differentiation, metabolism, regeneration, and cell death.^[5,6]^ The main targets of receptor TKIs are vascular endothelial growth factor receptor (VEGFR) 1 to 3, fibroblast growth factor receptor (FGFR) 1 to 4, platelet-derived growth factor receptor (FGFR) 1 to 4, platelet-derived growth factor α, c-Kit, RET, etc,^[7]^ in addition to enhancing the infiltration of NK cells into the tumor and their cytotoxicity, and promoting the expression of cytotoxic factors in the tumor tissues and thus exerting antitumor activity.^[8]^ In view of the multitargeted and multidirectional antitumor effects of multireceptor TKIs in HCC, combination therapy based on multireceptor TKIs has become the first choice for late-stage treatment, and also provides many new ideas for the treatment of HCC.^[9]^

Bibliometrics is a branch of informatics that analyzes literature from a quantitative and qualitative perspective by studying the systematic and bibliometric characteristics of literature^[10]^ to assess the status, trends, and frontiers of research activities^[10–12]^ This study retrieved bibliometric data (annual articles, countries/regions, authors, institutions, disciplines, journals, references, and keywords) on liver cancer and TKI research from various fields based on the Web of Science™ Core Collection (WoSCC), and descriptive statistics were analyzed. This paper discusses the current status, hotspots and cutting-edge literature of research on liver cancer and TKIs, and uses CiteSpace and VOSviewer to map the knowledge landscape to provide a reference for follow-up-related research.

## 2. Methods and materials

The WoSCC database has better accuracy in annotating literature types than any other database and is considered the best choice for literature analysis, so we chose to search in this database. We searched WOS on April 12, 2024, for all articles related to the use of tyrosinase inhibitors in HCC between 2004 and December 31, 2023, with the following search formula: ((((((TS=(Tyrosine Kinase Inhibitors)) OR TS=(Inhibitors, Tyrosine Kinase)) OR TS=(Kinase Inhibitors, Tyrosine)) OR TS=(Tyrosine Kinase Inhibitor)) OR TS=(TKI Tyrosine Kinase Inhibitors)) OR TS=(Tyrosine Protein Kinase Inhibitors)) OR TS=(TKI) AND (((((((((((((((((((((TS=(Liver Neoplasms)) OR TS=(Neoplasms, Hepatic)) OR TS=(Neoplasms, Liver)) OR TS=(Liver Neoplasm)) OR TS=(Neoplasm, Liver)) OR TS=(Hepatic Neoplasms)) OR TS=(Hepatic Neoplasm)) OR TS=(Neoplasm, Hepatic)) OR TS=(Cancer of Liver)) OR TS=(Hepatocellular Cancer)) OR TS=(Cancers, Hepatocellular)) OR TS=(Hepatocellular Cancers)) OR TS=(Hepatic Cancer)) OR TS=(Cancer, Hepatic)) OR TS=(Cancers, Hepatic)) OR TS=(Hepatic Cancers)) OR TS=(Liver Cancer)) OR TS=(Cancer, Liver)) OR TS=(Cancers, Liver)) OR TS=(Liver Cancers)) OR TS=(Cancer of the Liver)) OR TS=(Cancer, Hepatocellular). Literature screening for this study was based on the following inclusion criteria: (1) there were full-text publications related to the application of tyrosinase inhibitors in HCC; (2) the articles and review manuscript categories were written in English; and (3) the articles were published between January 1, 2004, and December 31, 2023. The exclusion criteria were as follows: (1) the topic was not related to tyrosinase inhibitors in HCC application; (2) the articles were conference abstracts, news, briefs, etc. Plain text versions of the papers were exported.

GraphPad Prism v8.0.2 was used to analyze and plot annual papers, national publication trends, and rates. In addition, CtieSpace (6.2.4R (64-bit) Advanced Edition) and VOSviewer (v.1.6.18) were used to analyze these data and visualize the scientific knowledge graph.

**Figure 1.**
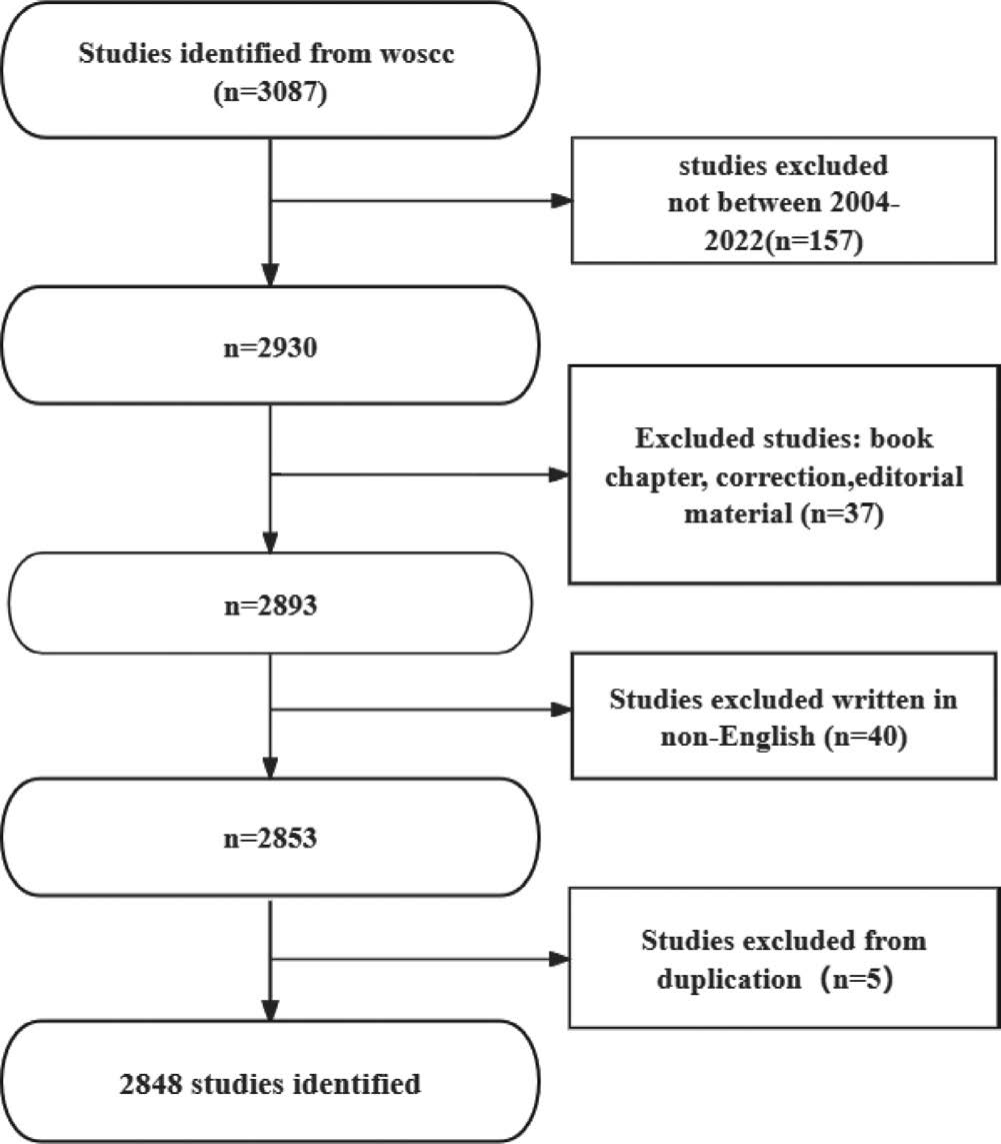
Flowchart of literature search.

VOSviewer v.1.6.17, created by Van Eck and Waltman^[13]^ in 2009, is a free JAVA-based software for analyzing large amounts of literature data and displaying it in a map format. To visualize the results of research in a particular field by mapping the co-citation network of literature, Professor Chaomei Chen created the CiteSpace (6.2.4R) software, which envisages the use of an experimental framework to study new concepts and evaluate existing technologies. This enables users to better understand the field of knowledge, research frontiers, and trends, and predict their future research progress.^[14]^ Figure 1 shows the search strategy and selection flowchart for this study.

## 3. Results

The results showed that from January 1, 2004, to December 31, 2023, the WoSCC database contained 2848 publications on the use of tyrosinase inhibitors in HCC, including 2151 articles and 697 reviews. The literature involves 80 countries and regions, 3265 institutions, and 16,653 authors.

**Figure 2.**
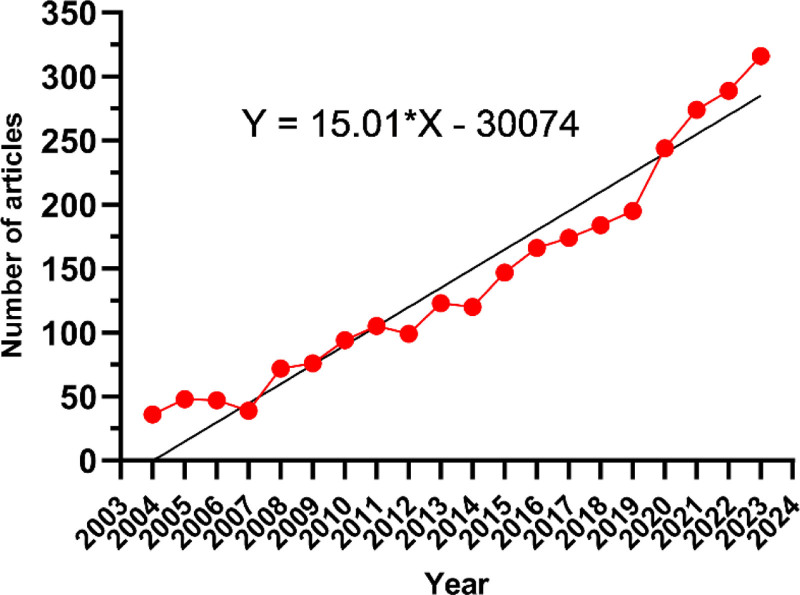
Annual volume of publications.

Since 2004, the number of papers published each year has slowly increased (Fig. 2). We divide it into 3 phases, 2004 to 2010 with a slow growth and less than 100 papers per year, which indicates that the field is not paid attention by researchers, 2011 to 2019 with a gradual increase in the number of papers, which indicates that the field gradually enters into the field of vision of researchers, and 2020 with a rapid increase in the number of papers after 2020 and reaching a peak in 2023, which indicates that the field receives wide attention after 2020. After 2020, the number of publications in this field increases rapidly and reaches a peak in 2023, indicating that this field is widely concerned after 2020.

### 3.1. Countries and institutions

**Figure 3.**
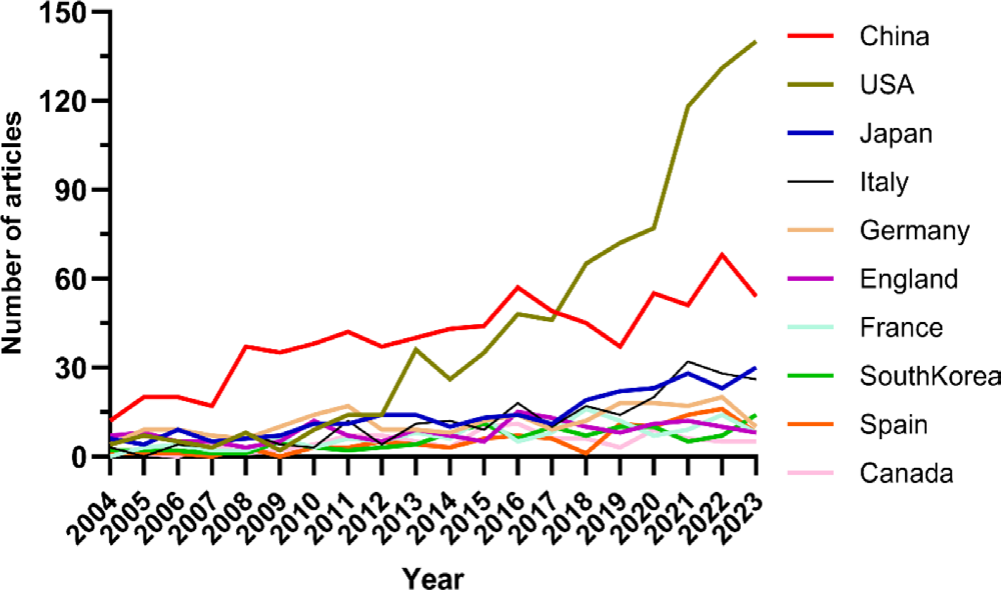
Line graph of national issuances.

**Figure 4.**
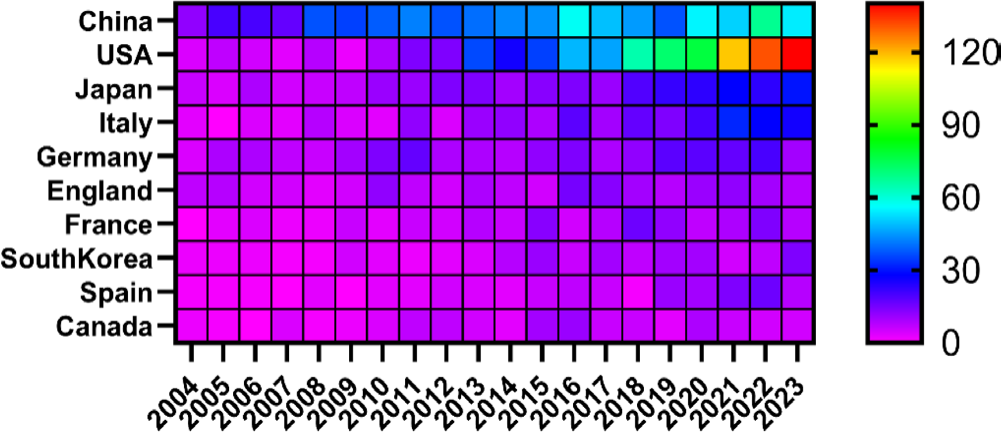
Heat map of national issuances.

Research on the application of tyrosinase inhibitors in liver cancer has been conducted in 80 countries and regions. Figures 3 and 4 show the annual publication volume of the top 10 countries in the past decade, and the top 5 countries in this field are China, the United States, Japan, Italy, and Germany. China and the United States accounted for 58.64% of the total number of publications, far exceeding other countries.

**Figure 5.**
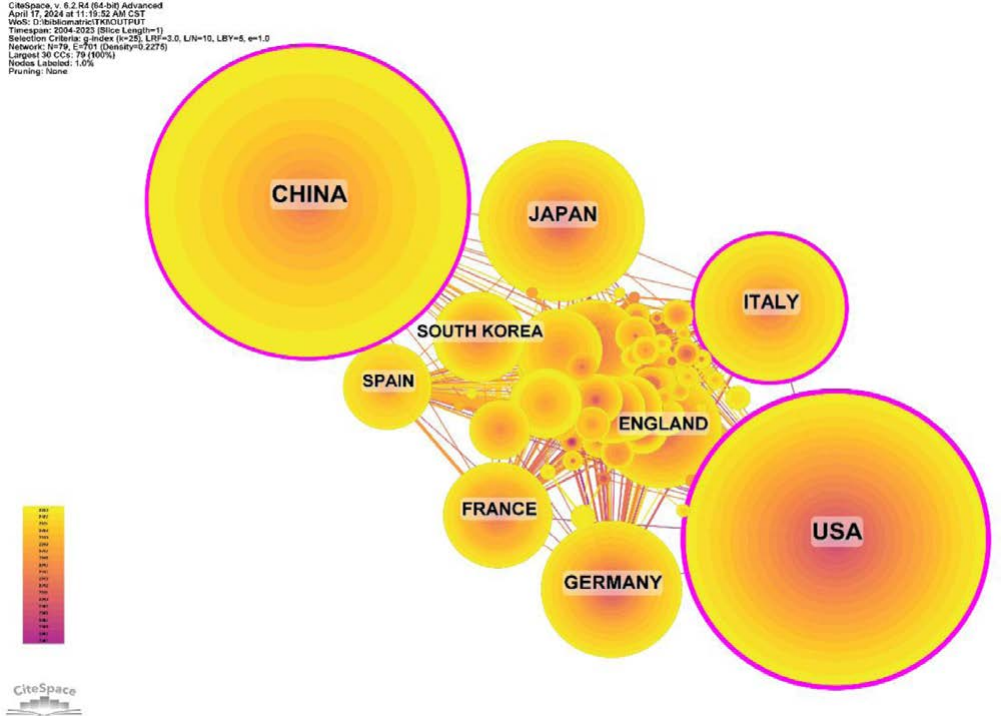
Country cooperation network diagram.

Among the top 10 countries/regions in terms of the number of published papers, the United States has 53,712 citations (Table 1), which is far more than all other countries/regions, and its citation/publication ratio (67.14) ranks high among all countries, indicating that the quality of its published papers is generally high. China is the 1st country in terms of the number of publications (870) and ranks 2nd in terms of the number of citations (29,464), and its citation/publication ratio (33.87) is ranked at the back of the list. The collaborative network is shown in Figure 5, with close cooperation between China, the most productive country, and the USA. The United States cooperates closely with Italy, Germany, and the United Kingdom, while China cooperates even more closely with Japan, Korea, and Spain. China not only has a large number of publications and a high citation frequency but also has a centrality of 0.12, indicating that it is currently the leading country in this field.

**Table 1:** National communications scale.

Rank	Country/region	Article counts	Centrality	Percentage (%)	Citation	Citation per publication
1	China	870	0.12	30.55	29,464	33.87
2	USA	800	0.35	28.09	53,712	67.14
3	Japan	280	0	9.83	19,541	69.79
4	Italy	238	0.16	8.36	18,477	77.63
5	Germany	232	0.1	8.15	16,805	72.44
6	England	166	0.06	5.83	13,145	79.19
7	France	138	0.05	4.85	13,975	101.27
8	South Korea	113	0.02	3.97	9303	82.33
9	Spain	103	0.07	3.62	10,142	98.47
10	Canada	97	0.04	3.41	9624	99.22

**Figure 6.**
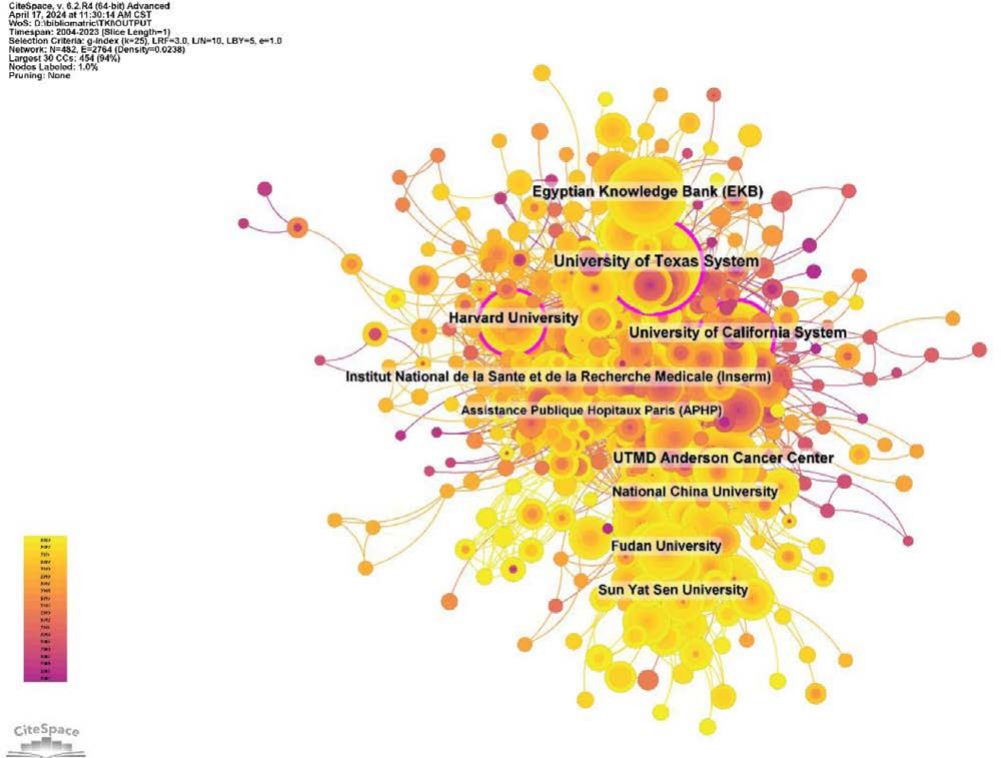
Institutional collaborative network map.

A total of 3265 institutions systematically published articles on the use of tyrosinase inhibitors in HCC. Among the top 10 institutions in terms of publications, 4 were from the United States, 3 were from China, 2 were from France, and 1 was from Egypt (Table 2 and Fig. 6). The University of Texas System published the most literature (96 papers, 10,261 citations, and 106.89 citations/paper). Egyptian Knowledge Bank (76 papers, 1460 citations, and 19.21 citations/paper) ranked second, UTMD Anderson Cancer Center (74 papers, 7542 citations, and 101.92 citations/paper) ranked third, and Harvard University (73 papers, 10,795 citations, and 147.88 citations/paper) ranked fourth. After further analysis, we found that domestic and foreign institutions prefer to cooperate with their own domestic units, so we call for strengthening cooperation between domestic and foreign institutions and breaking academic barriers.

**Table 2:** Institutional issuance form.

Rank	Institution	Country	Number of studies	Total citations	Average citation
1	University of Texas System	USA	96	10,261	106.89
2	Egyptian Knowledge Bank (EKB)	Egypt	76	1460	19.21
3	UTMD Anderson Cancer Center	USA	74	7542	101.92
4	Harvard University	USA	73	10,795	147.88
5	University of California System	USA	70	6274	89.63
6	Institut National de la Sante et de la Recherche Medicale (Inserm)	France	58	4774	82.31
7	National China University	China	53	3940	74.34
8	Sun Yat Sen University	China	51	2458	48.20
9	Fudan University	China	47	1084	23.06
10	Assistance Publique Hopitaux Paris (APHP)	France	43	4706	109.44

### 3.2. Journals

**Figure 7.**
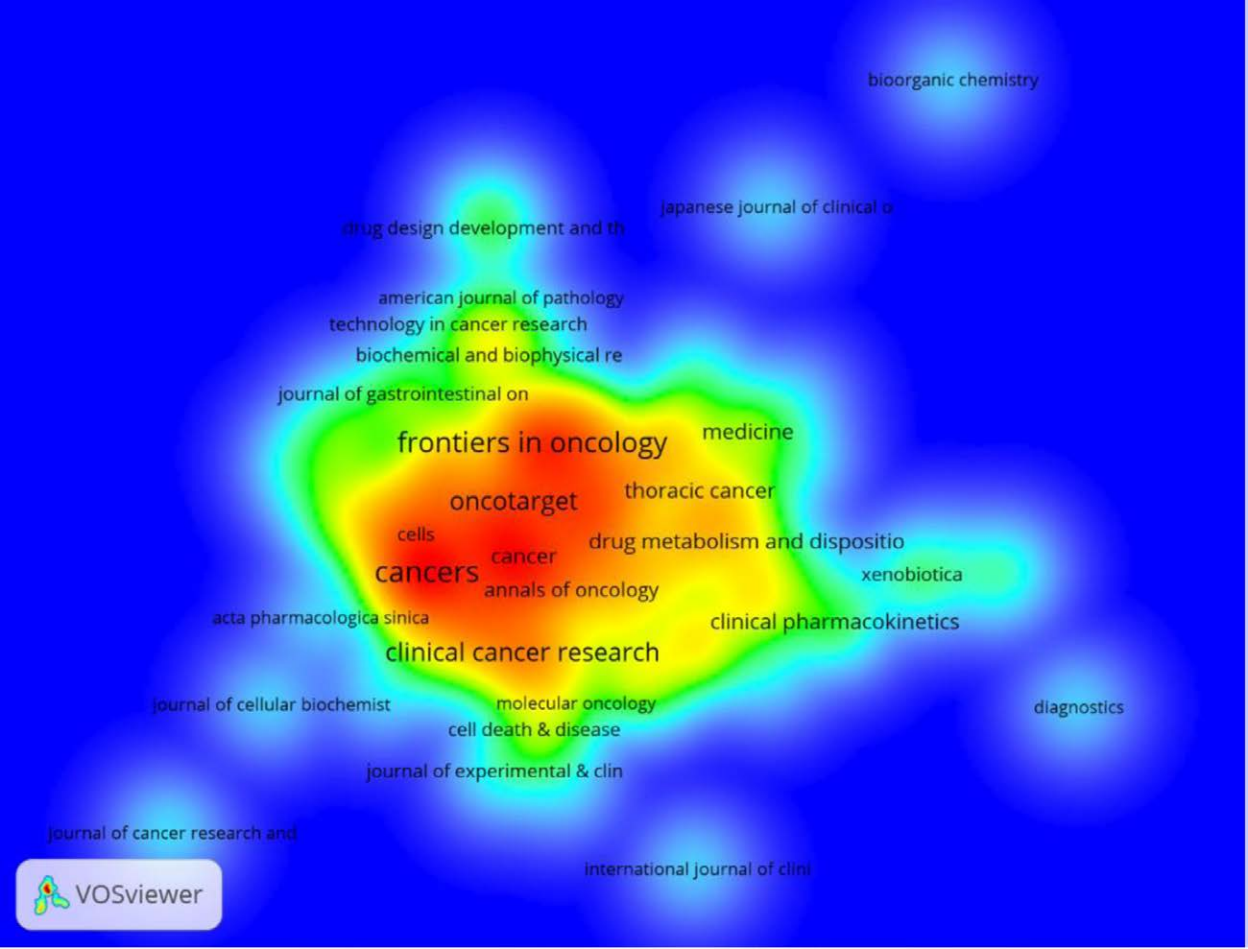
Density map of magazine issuance.

The top 10 most productive and cited journals are listed in Tables 3 and 4. Figure 7 shows the density statistics of magazine circulation. Cancers (79 articles, 2.77%) is the most published journal in the field, followed by Frontiers in Oncology (67 articles, 2.35%), Clinical Cancer Research (47 articles, 1.65%), and Oncotarget (41 articles, 1.44%). Among the top 10 most prolific journals, Clinical Cancer Research had the highest IF of 11.5. A total of 90% of these journals were classified in Q1 or Q2.

**Table 3:** Scale of magazine publications.

Rank	Journal	Article counts	Percentage (2848)	IF	Quartile in category
1	Cancers	79	2.77	5.2	Q2
2	Frontiers in Oncology	67	2.35	4.7	Q2
3	Clinical Cancer Research	47	1.65	11.5	Q1
4	Oncotarget	41	1.44	5.1	Q2
5	Plos One	41	1.44	3.7	Q2
6	Molecular Cancer Therapeutics	34	1.19	5.7	Q2
7	BMC Cancer	32	1.12	3.8	Q2
8	Cancer Chemotherapy and Pharmacology	32	1.12	3.0	Q3
9	International Journal of Molecular Sciences	32	1.12	5.6	Q1
10	Frontiers in Pharmacology	31	1.09	5.6	Q1

**Table 4:** Journal co-citation table.

Rank	Cited Journal	Co-Citation	IF (2022)	Quartile in category
1	J Clin Oncol	1833	45.4	Q1
2	Clin Cancer Res	1820	11.5	Q1
3	New Engl J Med	1688	158.5	Q1
4	Cancer Res	1616	11.2	Q1
5	Lancet Oncol	1166	51.1	Q1
6	Ann Oncol	1058	50.5	Q1
7	Lancet	1051	168.9	Q2
8	Brit J Cancer	1036	8.8	Q1
9	Oncogene	975	8.0	Q1
10	Hepatology	966	14.0	Q1

**Figure 8.**
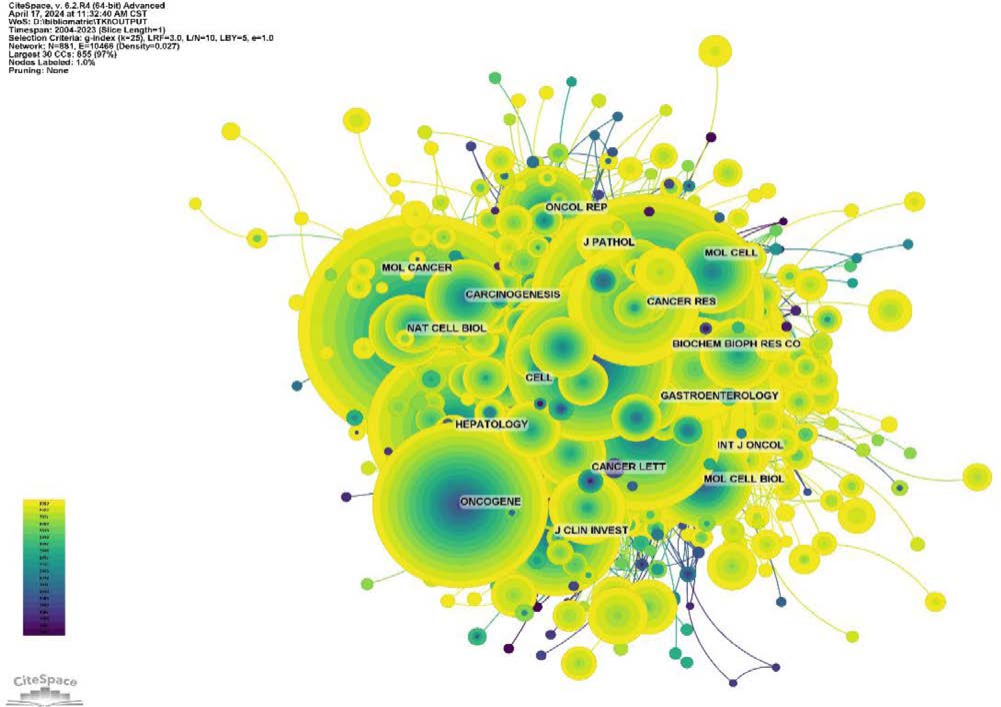
Journal co-citation network diagram.

Journal impact is determined by how often it is co-cited, which indicates whether the journal has had a significant impact on the scientific community. According to Figure 8 and Table 4, the journal with the highest number of co-citations is J Clin Oncol (1833), followed by Clin Cancer Res (1820), and New Engl J Med (1688). Among the top 10 most co-cited journals, Lancet was cited 1051 times with the highest IF among the top 10 journals (168.9). Of the journals that were co-cited, 60% of the journals were in the Q1/Q2 region.

**Figure 9.**
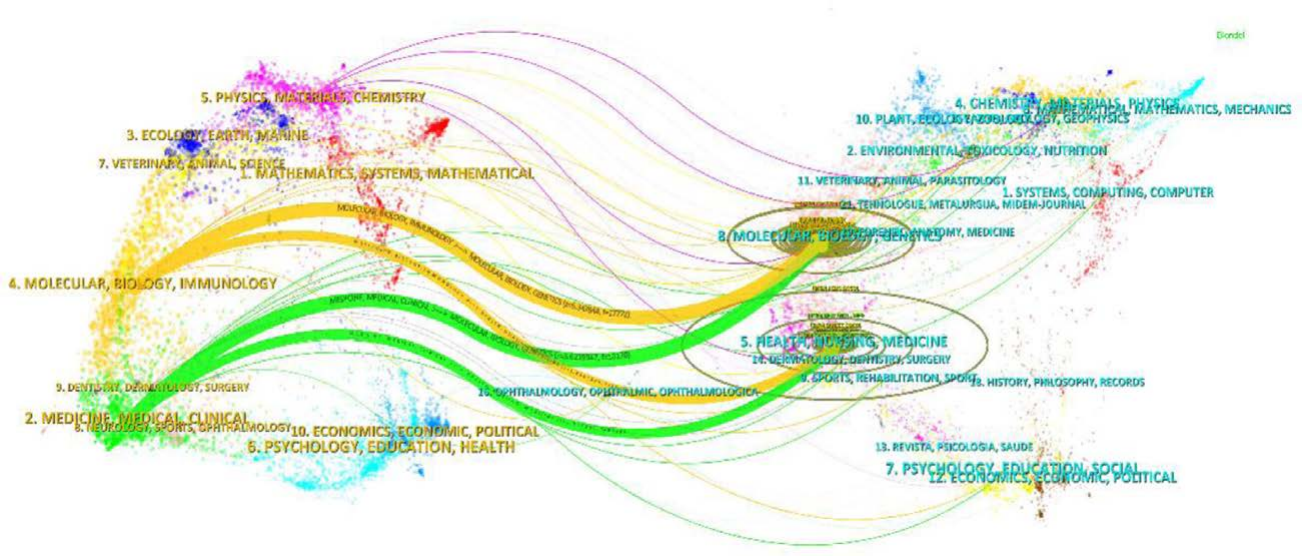
Double-stacked map of journals.

The thematic distribution of academic publications is shown by a double map overlay (Fig. 9). Colored tracks indicate citation links, with citing journals on the left and cited journals on the right. Based on the displayed results, we identified 4 main colored citation paths, that is, studies published in journals in the field of molecular/biology/immunology were mainly cited by studies published in journals in the fields of molecular/biology/genetics and health/nursing/medicine, while studies published in journals in the field of medicine/medical/clinical were mainly cited by studies published in journals in the fields of molecular/biology/genetics and health/nursing/medicine.

### 3.3. Authors and co-citing authors

**Figure 10.**
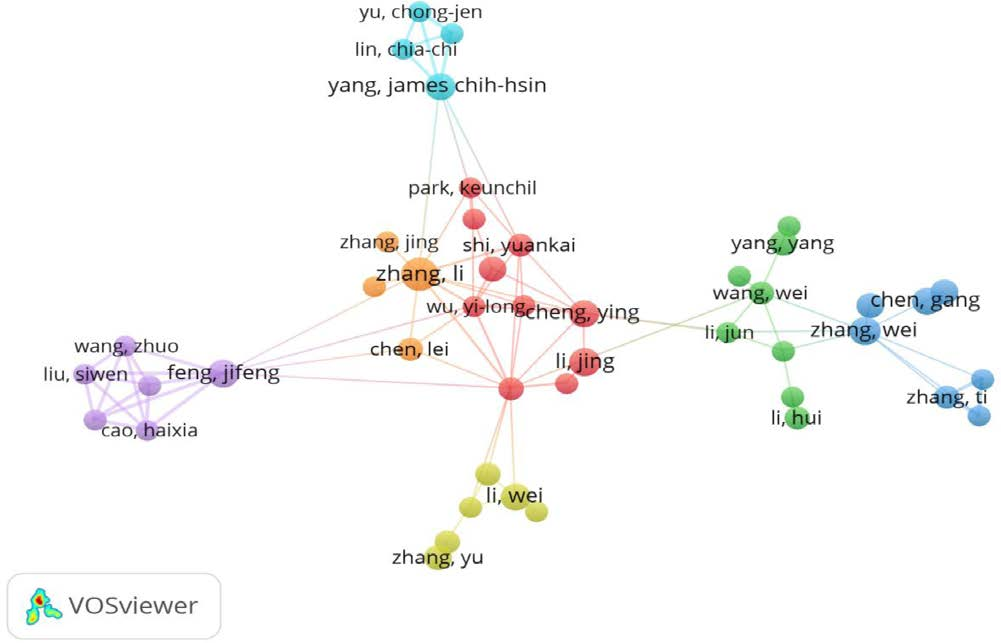
Author collaboration network.

Of all the authors who have published literature related to the use of tyrosinase inhibitors in HCC, the top 10 authors who have published the most papers are listed in Table 5. The top 10 authors published a total of 129 papers, which is 4.53% of all the papers in the field. Attwa MW published the most research papers with 20 papers, followed by Kadi AA (19 papers), Cheng AL (15 papers), and Kudo M (14 papers). Further analysis showed that 5 of the top 10 authors were from China, 3 from Saudi Arabia, 1 from Japan, and 1 from Spain. CiteSpace visualized the network between authors (Fig. 10).

**Table 5:** Table of author publications and co-citations.

Rank	Author	Count	Location	Rank	Co-cited author	Citation
1	Attwa MW	20	Saudi Arabia	1	Llovet JM	726
2	Kadi AA	19	Saudi Arabia	2	Kudo M	411
3	Cheng AL	15	China	3	Bruix J	386
4	Kudo M	14	Japan	4	Zhu AX	378
5	Zhang L	12	China	5	Cheng AL	376
6	Chen KF	10	China	6	Abou-Alfa GK	329
7	Darwish HW	10	Saudi Arabia	7	Finn RS	311
8	Marin JJG	10	Spain	8	Wilhelm SM	242
9	Zhu AX	10	China	9	Jemal A	225
10	Feng JF	9	China	10	Motzer RJ	220

**Figure 11.**
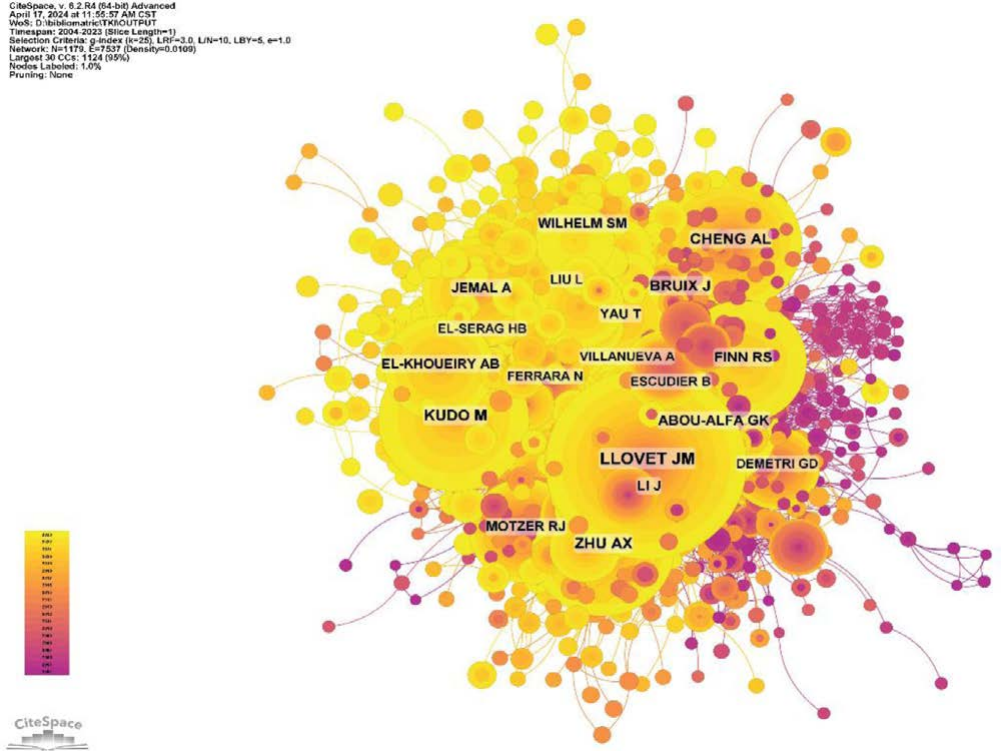
Author co-citation network map.

Figure 11 and Table 5 show the top 10 authors with the highest number of co-citations and citations, respectively. Ninety-three authors were cited more than 20 times in total, indicating the high reputation and impact of their research. The largest nodes are associated with the most co-cited authors, including Llovet JM (726 citations), Kudo M (411 citations), and Bruix J (386 citations).

### 3.4. Co-cited references

**Figure 12.**
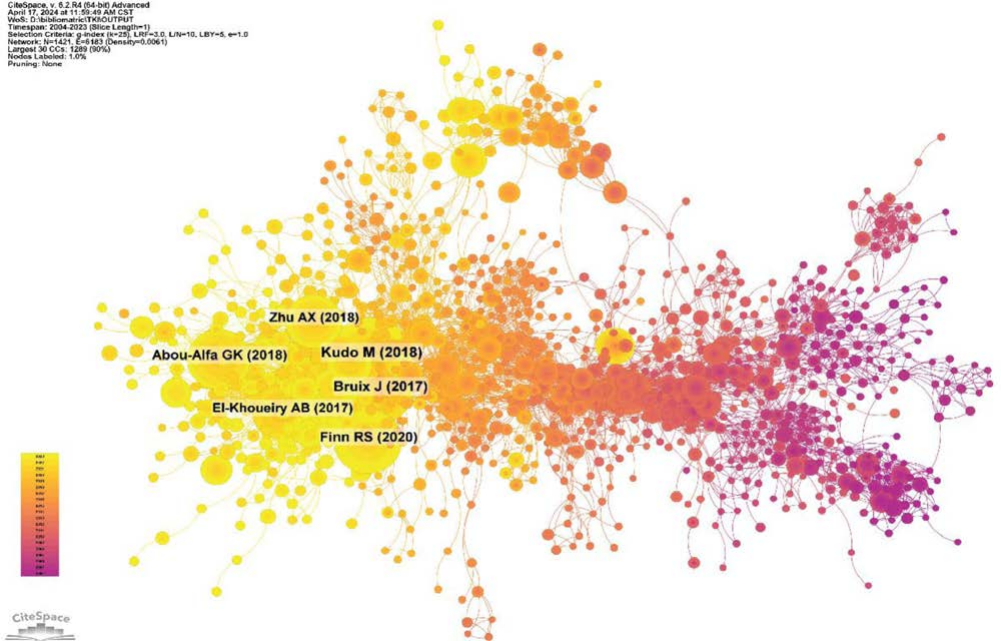
Network diagram of co-cited literature.

Taking a 1-year time slice with a time horizon of 2004 to 2023, the co-cited references network has 1421 nodes and 6183 links (Fig. 12). According to the top 10 most co-cited articles (Table 6), LANCET’s “ Lenvatinib versus sorafenib in first-line treatment of patients with unresectable HCC: a randomized phase 3 noninferiority trial,” the first author of the article is Kudo M, which is based on a phase 2 trial in which levatinib, an inhibitor of VEGFR 1 to 3, FGFR 1 to 4, PDGF receptor a, RET and KIT, showed activity against HCC. This was an open-label, phase 3, multicenter, noninferiority trial that enrolled patients with unresectable HCC who had not received advanced treatment at 154 sites in 20 countries across Asia-Pacific, Europe, and North America. Patients were randomly assigned (1:1) via an interactive voice-to-web response system—with region, gross portal vein invasion, extrahepatic spread or both, Eastern Cooperative Oncology Group performance status and body weight as stratification factors—to receive oral lenvatinib (12 mg/day for those weighing greater than or equal to 60 kg and 8 mg/day for those weighing less than 60 kg) or sorafenib 400 mg twice daily The cycle was 28 days. The primary endpoint was overall survival (OS) from the date of randomization to the date of death from any cause. Efficacy analyses followed the intention-to-treat principle, and only patients who received treatment were included in safety analyses. The noninferiority margin was set at 1.08. A total of 1492 patients were recruited between March 1, 2013, and July 30, 2015. A total of 954 eligible patients were randomly assigned to lenvatinib (478) or sorafenib (476). The median survival time for lenvatinib was 13.6 months (95% CI: 12.1–14.9), which was not inferior to sorafenib (12.3 months, 10.4–13.9; hazard ratio 0.92, 95% CI: 0.79–1.06) and met the criterion for noninferiority. The most common adverse events of any grade with lenvatinib were hypertension (201 [42%]), diarrhea (184 [39%]), decreased appetite (162 [34%]), and weight loss (147 [31%]); the most common adverse events of any grade with sorafenib were palmoplantar erythromelalgia (249 [52%]), diarrhea (220 [46%]), hypertension (144 [30%]), and decreased appetite (127 [27%]).^[15]^

**Table 6:** Co-cited literature table.

Rank	Title	Journal	Author(s)	Total citations
1	Lenvatinib versus sorafenib in first-line treatment of patients with unresectable hepatocellular carcinoma: a randomized phase 3 non-inferiority trial	Lancet	Kudo M	298
2	Regorafenib for patients with hepatocellular carcinoma who progressed on sorafenib treatment (RESORCE): a randomized, double-blind, placebo-controlled, phase 3 trial	Lancet	Bruix J	201
3	Atezolizumab plus Bevacizumab in Unresectable Hepatocellular Carcinoma	New England Journal of Medicine	Finn RS	196
4	Cabozantinib in Patients with Advanced and Progressing Hepatocellular Carcinoma	New England Journal of Medicine	Abou-Alfa GK	177
5	Nivolumab in patients with advanced hepatocellular carcinoma (CheckMate 040): an open-label, non-comparative, phase 1/2 dose escalation and expansion trial	Lancet	El-Khoueiry AB	146
6	Pembrolizumab in patients with advanced hepatocellular carcinoma previously treated with sorafenib (KEYNOTE-224): a non-randomized, open-label phase 2 trial	Lancet Oncology	Zhu AX	140
7	Ramucirumab after sorafenib in patients with advanced hepatocellular carcinoma and increased α-fetoprotein concentrations (REACH-2): a randomized, double-blind, placebo-controlled, phase 3 trial	Lancet Oncology	Zhu AX	113
8	Pembrolizumab As Second-Line Therapy in Patients With Advanced Hepatocellular Carcinoma in KEYNOTE-240: A Randomized, Double-Blind, Phase III Trial	Journal of Clinical Oncology	Finn RS	108
9	Sorafenib in advanced hepatocellular carcinoma	New England Journal of Medicine	Llovet JM	103
10	EASL Clinical Practice Guidelines: Management of hepatocellular carcinoma	Journal of Hepatology	European Assoc Study Liver	89

The second highest was published in LANCET, “Regorafenib for patients with HCC who progressed on sorafenib treatment (RESORCE): a randomized, double-blind, placebo-controlled, phase 3 trial,” authored by Bruix J, which argues that in the context of no systemic treatment currently available for patients with HCC whose disease progressed on sorafenib treatment, the study is a good example of how to improve the quality of care for patients with HCC. We aimed to evaluate the efficacy and safety of regorafenib in patients with HCC whose disease progressed during sorafenib treatment. In this randomized, double-blind, parallel-group, phase 3 trial, 152 sites in 21 countries recruited adult patients with HCC who were able to tolerate sorafenib (≥400 mg/day for ≥20 days during the last 28 days of treatment), whose disease progressed during sorafenib treatment and who had Child-Pugh A liver function. Using a computer-generated randomization list and an interactive voice response system, and based on geographic region, Eastern Cooperative Oncology Group performance status, macrovascular invasion, extrahepatic disease, and alpha-fetoprotein levels, the researchers randomly assigned participants (2:1) to optimal supportive care plus oral regorafenib 160 mg or placebo once daily during weeks 1 to 3 of each 4-week cycle. Investigators, patients, and funders were unaware of the treatment assignment. The primary endpoint was OS (defined as the time from random allocation to death from any cause) and was analyzed by intention to treat. The trial is registered with ClinicalTrials.gov as NCT01774344. Between May 14, 2013, and December 31, 2015, 843 patients were screened, of whom 573 were enrolled and randomly assigned (379 received regorafenib and 194 received placebo; efficacy analyzed population), and 567 started treatment (374 treated with regorafenib and 193 with placebo; safety analysis population). Regorafenib improved OS with a hazard ratio of 0.63 (95% CI: 0.50–0.79; one-sided *P* < .0001); median survival was 10.6 months (95% CI: 9.1–12.1) for regorafenib versus 7.8 months (6.3–8.8) for placebo. Adverse events were reported in all regorafenib subjects (374 of 374 [100%]) and 179 of 193 placebo subjects (93%). The most common clinically relevant grade 3 or 4 treatment emergencies were hypertension (57 patients [15%] in the regorafenib group vs 9 patients [5%] in the placebo group), skin reactions of the hands and feet (47 patients [13%] vs 1 patient [1%]), fatigue (34 patients [9%] vs 9 patients [5%]), and diarrhea (12 patients [3%] vs none). Of the 88 deaths (grade 5 adverse events) reported during the study period (50 patients [13%] treated with regorafenib and 38 patients [20%] treated with placebo), the investigators attributed 7 (2%) in the regorafenib group and 2 (1%) in the placebo group to the study medication, with hepatic failure occurring in 2 patients (1%) in the placebo group. Regorafenib is the only systemic therapy that provides a survival benefit for patients with HCC whose disease has progressed after sorafenib treatment. Future trials should explore combinations of regorafenib with other systemic agents, as well as third-line therapy for patients who have failed or are intolerant to sorafenib and regorafenib.^[16]^

**Figure 13.**
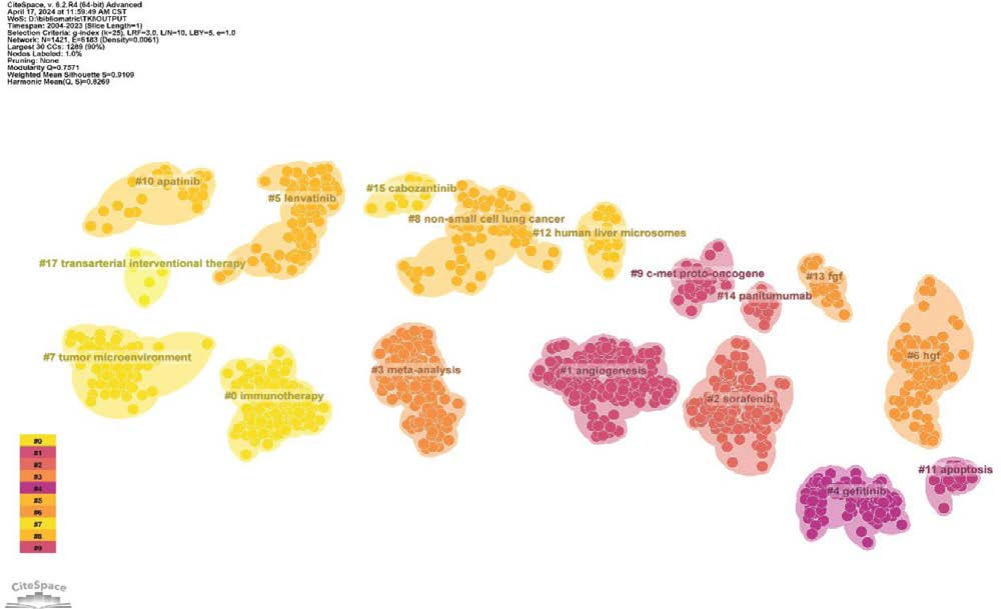
Clustering map of co-cited literature.

**Figure 14.**
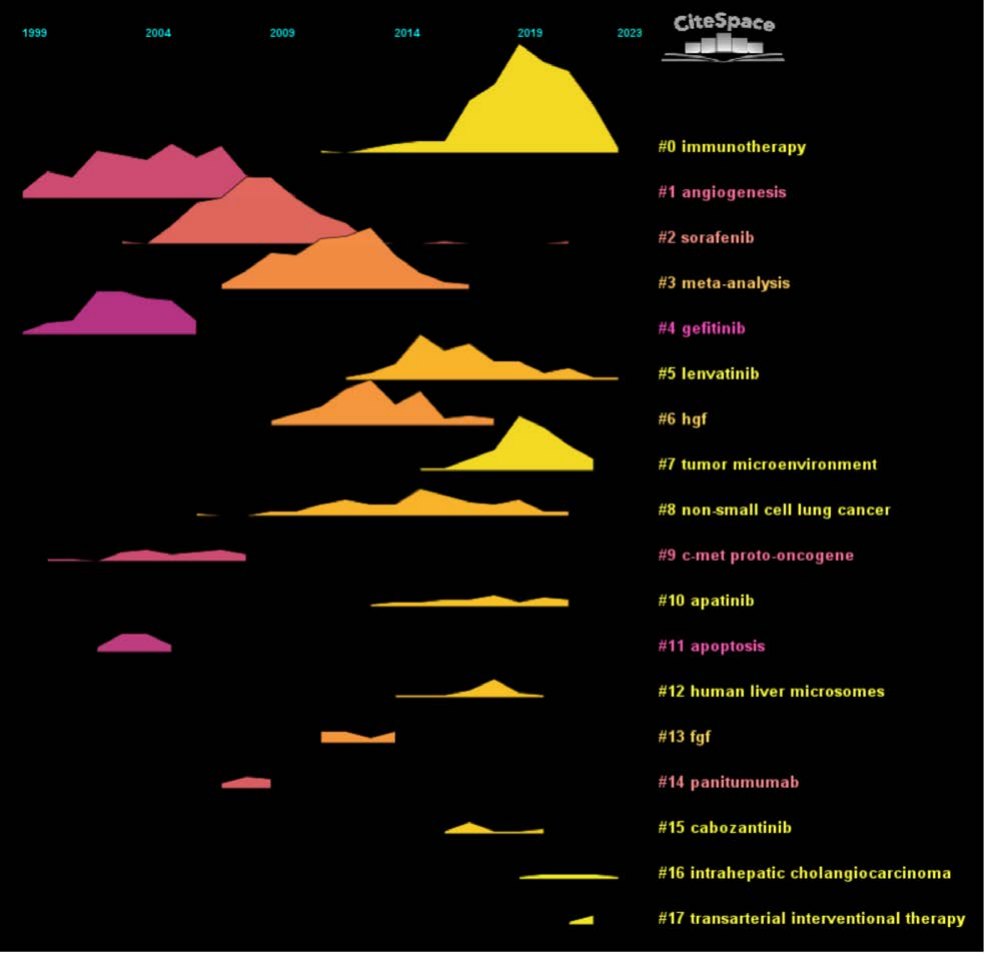
Co-cited literature volcano map.

We performed co-citation reference clustering and temporal clustering analyses (Figs. 13 and 14). We found that angiogenesis (cluster1), gefitinib (cluster4), c-met proto-oncogene (cluster9), and apoptosis (cluster11) were early research hotspots. Sorafenib (cluster2), meta-analysis (cluster3), hgf (cluster6), fgf (cluster13), and panitumumab (cluster14) are hotspots for mid-stage research. Immunotherapy (cluster0), lenvatinib (cluster5), tumor microenvironment (cluster7), nonsmall cell lung cancer (cluster8), apatinib (cluster10), human liver microsomes (cluster12), cabozantinib (cluster15), intrahepatc cholangiocarcinoma (cluster16), and transarterial interventional therapy (cluster17) are hot topics and trends in the field.

### 3.5. Keyword analysis

**Figure 15.**
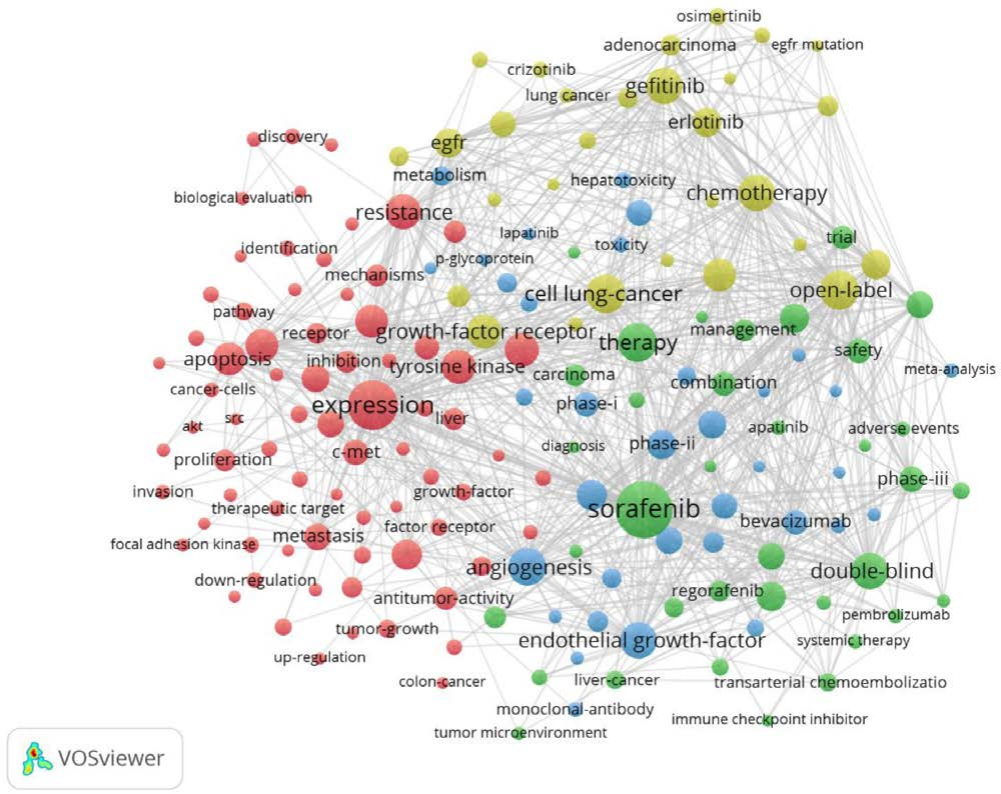
High-frequency keyword network map.

**Figure 16.**
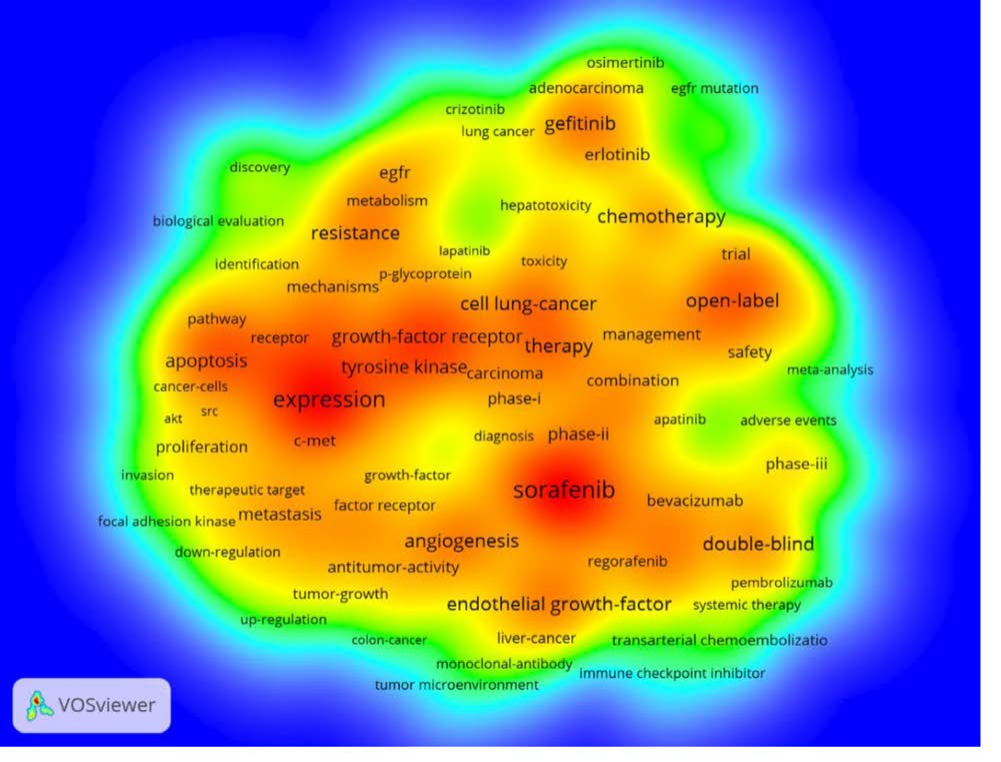
Keyword density chart.

**Figure 17.**
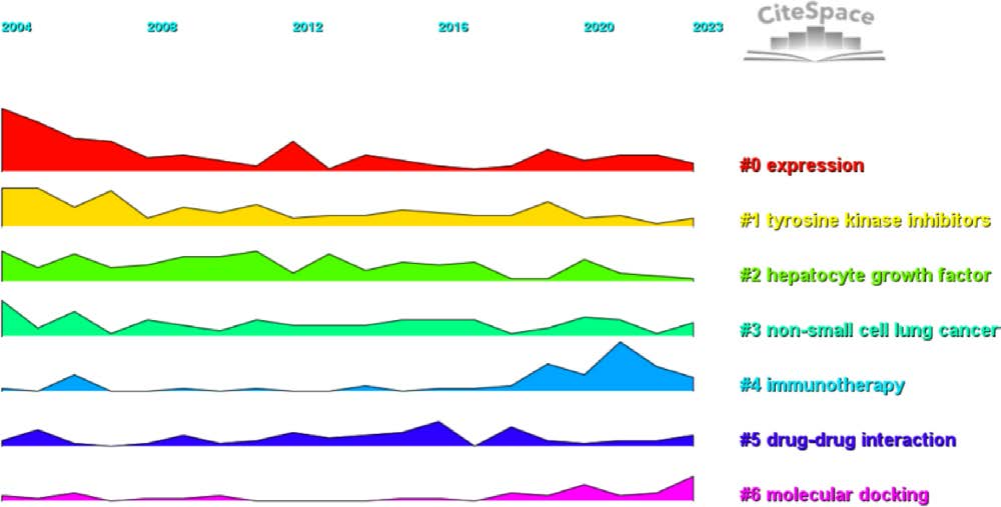
Keyword clustering volcano map.

**Figure 18.**
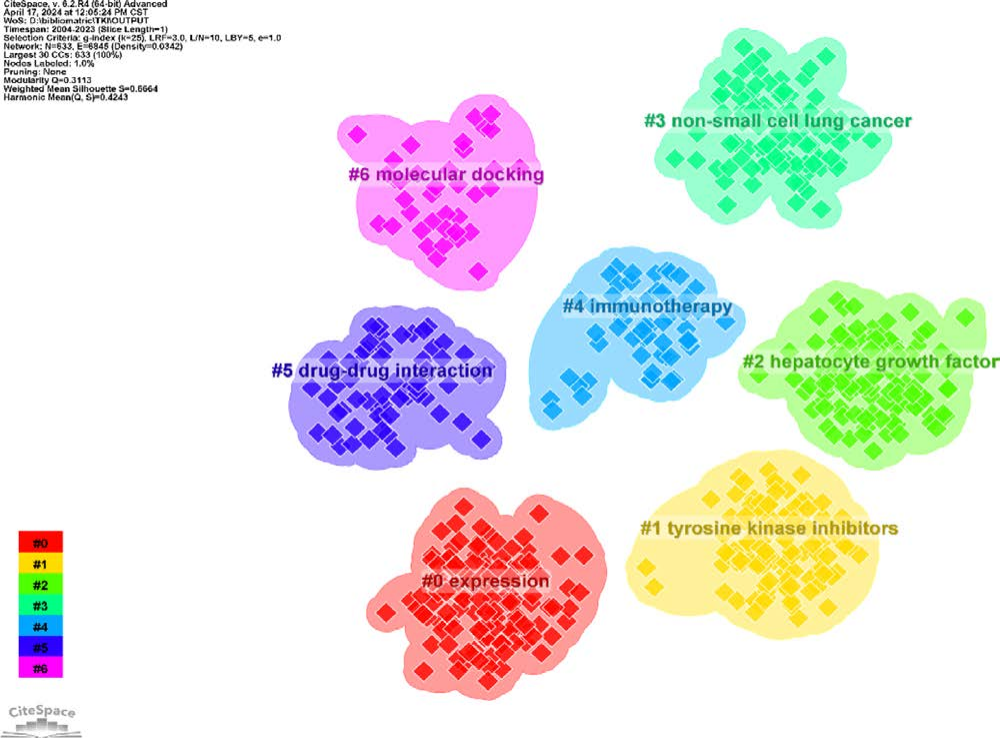
Keyword clustering map.

By analyzing keywords, we can quickly understand the situation and development direction of a field. According to the co-occurrence of keywords in VOSwiever, the most popular keyword was sorafenib (501), followed by expression (389), therapy (246), open-label (242), and angiogenesis (240) (Table 7 and Figs. 15 and 16). We removed useless keywords and constructed a network containing 174 keywords with at least 28 occurrences, yielding a total of 4 different clusters. Cluster 1 (red) had 74 keywords including expression, growth, pathway, protein, c-met, apoptosis, autophagy, design, factor receptor, discovery, invasion, migration, stat3, resistance, target, protect, mechanism, overexpression, potent, proliferation, and oxidative stress. Group 2 (green) has 36 keywords, including adverse events, blockade, combination, diagnosis, efficacy, immune checkpoint inhibitor, management, prognosis, resection, safety, systemic therapy, tumor microenvironment, and 1st-line therapy. Group 3 contains 35 keywords (in blue) including advanced hepatocellular carcinoma, angiogenesis, dasatinib, imatinib, meta analysis, hepatoxicity, pharmacokinetics, toxicity, vegf, and p-glycoprotein. Group 4 contains 29 keywords (yellow), including survival, chemotherapy, mutation, gene, acquired resistance, cisplatin, open label, survival, sensitivity, and Japanese patient. We plotted a volcano map via CiteSpace to visualize the research hotspots over time (Figs. 17 and 18).

**Table 7:** Table of high-frequency keywords.

Rank	Keyword	Counts	Rank	Keyword	Counts
1	Sorafenib	501	11	Growth-factor receptor	209
2	Expression	389	12	Inhibitor	190
3	Therapy	246	13	Tyrosine kinase	187
4	Open-label	242	14	Survival	185
5	Angiogenesis	240	15	Growth	180
6	Double-blind	223	16	Apoptosis	176
7	Chemotherapy	220	17	Activation	175
8	Endothelial growth-factor	217	18	Targeted therapy	173
9	Gefitinib	214	19	Efficacy	155
10	Resistance	214	20	Erlotinib	151

### 3.6. Co-cited references and keywords

**Figure 19.**
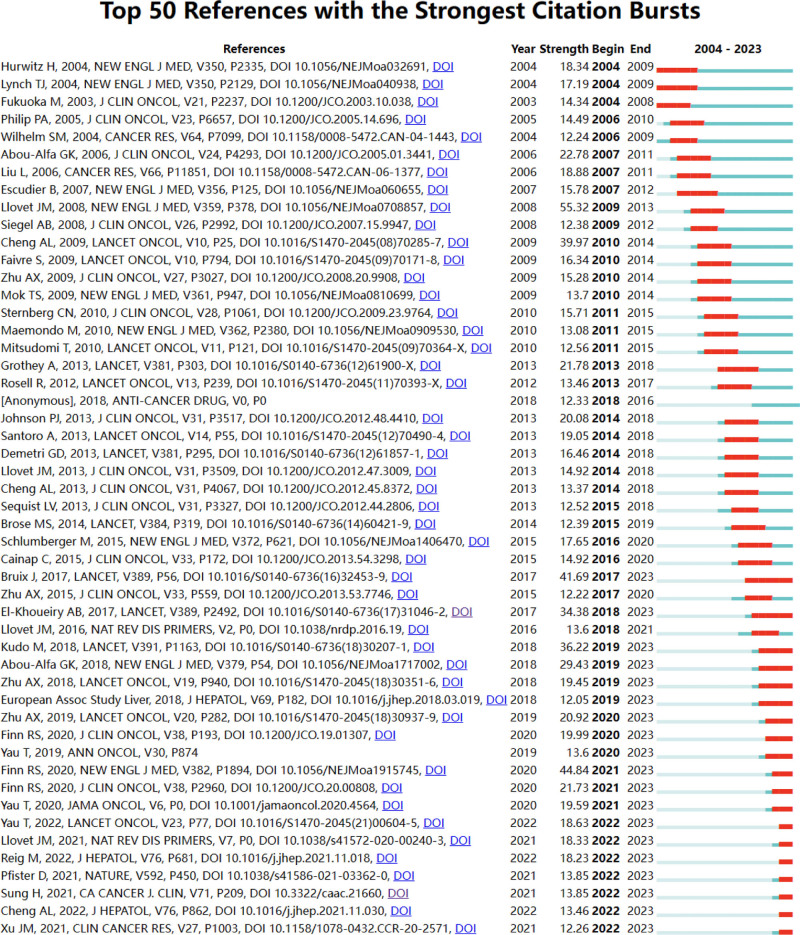
Cited literature emergence map.

Using CiteSpace, we came up with the 50 most reliable citation bursts for tyrosinase inhibitors in liver cancer applications. One of the most cited references (55.32) is “Sorafenib in Advanced Hepatocellular Carcinoma” published in The New England Journal of Medicine homepage, which was first authored by Llovet JM, the article concluded that there is no effective systemic therapy for patients with advanced HCC. In this multicenter, phase III, double-blind, placebo-controlled trial, we randomly assigned 602 patients with advanced HCC who had not received prior systemic therapy to receive sorafenib (at a dose of 400 mg twice daily) or placebo. The primary outcomes were OS and time to symptom progression. In the second planned interim analysis, there were 321 deaths and the study was stopped as a result. Median OS was 10.7 months in the sorafenib group and 7.9 months in the placebo group (hazard ratio 0.69 in the sorafenib group; 95% confidence interval 0.55–0.87; *P* < .001). The median time to symptom progression was not significantly different between the 2 groups (4.1 and 4.9 months, respectively; *P* = .77). The median time to radiological progression was 5.5 months in the sorafenib group and 2.8 months in the placebo group (*P* < .001). Seven patients (2%) in the sorafenib group and 2 patients (1%) in the placebo group showed partial response, and no patients showed complete response. In patients with advanced HCC, median survival and time to radiological progression were nearly 3 months longer in patients treated with sorafenib than in those treated with placebo (ClinicalTrials.gov number: NCT00105443). Forty-nine of the 50 references were published between 2004 and 2023, suggesting that these papers have been cited frequently over the last 20 years. Importantly, 19 of these papers are currently at peak citation (Fig. 19), implying that tyrosinase inhibitors in liver cancer will continue to be of interest in the future.

**Figure 20.**
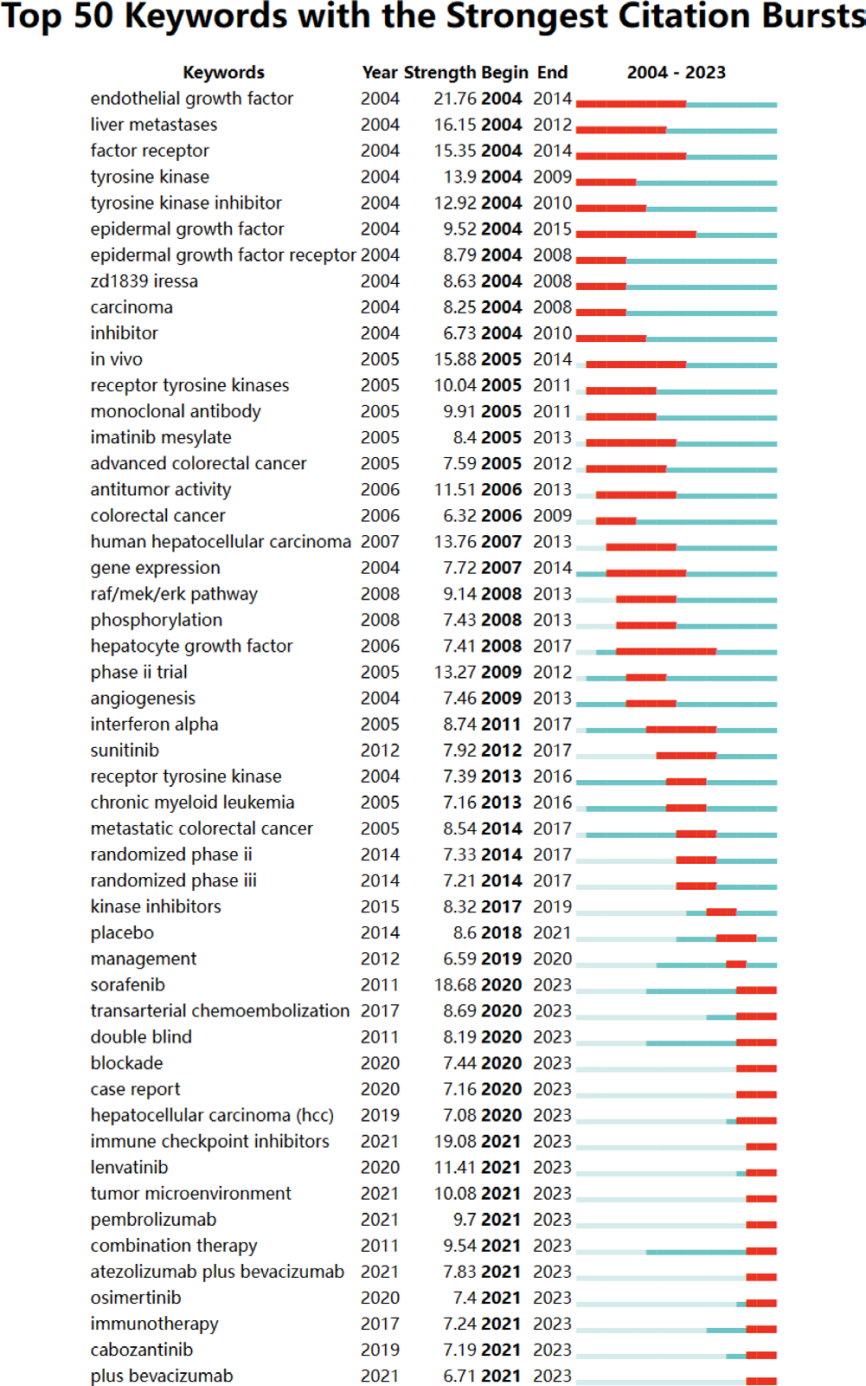
Keyword emergence map.

Among the 659 strongest mutated keywords in the field, we focused on the 50 keywords with the strongest mutations (Fig. 20), which represent the current research hotspots in the field and represent possible future research directions.

## 4. Discussion

In recent years, the incidence and mortality of primary liver cancer have been gradually increasing. Most patients with HCC are already in the middle and advanced stages when diagnosed, losing the chance of surgery, and the survival prognosis is poor, and systemic therapy and local therapy are the main treatment options for middle and advanced HCC.^[17]^

The progression of HCC is associated with an abnormal microenvironment of hepatocyte regeneration.^[18]^ The development and progression of HCC involves a combination of factors, especially chronic viral hepatitis and nonviral hepatitis; among them, hepatitis B virus and hepatitis C virus are the main causes of cirrhosis and the progression to HCC. In addition, obesity, alcoholism, diabetes mellitus, and dyslipidemia, which can cause inflammatory reactions or hepatic steatosis, are all etiological factors inducing the development of HCC.^[19–21]^

Multi-TKIs have been used in the treatment of HCC since 2007, and in the 2007 SHARP trial evaluating sorafenib for the treatment of HCC, patients in the placebo group had an OS of 7.9 months, which was prolonged to 10.7 months in the sorafenib group.^[22]^ The drug’s safety and moderate efficacy were validated in patients in the Asia-Pacific region, which led to the approval of sorafenib as a first-line agent for the treatment of advanced unresectable HCC.^[23]^ In 2018, The Lancet published a phase III clinical study of lenvatinib versus sorafenib for the first-line treatment of unresectable HCC (the REFLECT study). The results showed that the OS of lenvatinib treatment group was noninferior to that of sorafenib, while it was significantly better than sorafenib in terms of progression-free survival (PFS), time to disease progression, and objective remission rate (ORR). Moreover, the efficacy of lenvatinib in Chinese patients with HCC, especially hepatitis B virus-associated HCC, was significantly better than sorafenib, with ORR, PFS, time to disease progression, and OS being 2.6-fold, 2.6-fold, 3.0-fold, and 1.5-fold higher than that of sorafenib, respectively, suggesting that lenvatinib is more suitable for Chinese patients with HCC.^[15]^ Lenvatinib mainly delays HCC progression by inhibiting VEGFRs (VEGFR1, VEGFR2, and VEGFR3) and FGFRs (FGFR1, FGFR2, FGFR3, and FGFR4). The results of the LEAP-002 study presented at the European Society for Medical Oncology Congress 2022^[24]^ showed that the median OS of the lenvatinib treatment group reached 19.0 months, which is the longest median OS for single-agent, first-line treatment of unresectable HCC at this point in time; and the ORR (mRECIST by BICR) of 34.1% also refreshed the efficacy data for single-agent treatment.

In addition, multi-TKIs such as lenvatinib can release the immunosuppressive effect caused by the vascular endothelial growth factor pathway, and release the inhibitory effect on the secretion of interferon-γ and granzyme B, thus enhancing the killing effect of cytotoxic T cells and the presentation effect of antigen-presenting cells.^[25,26]^ On the other hand, lenvatinib can enhance the efficacy of programmed cell death 1 (PD-1) inhibitors by targeting FGFR 4 to reduce the level of programmed cell death receptor-ligand 1 in HCC cells, and regulate the differentiation of T cells.^[27,28]^ HCC-derived CSF1 can convert macrophages to an M2 phenotype to drive immune escape and anti-PD-1 tolerance,^[29]^ but lenvatinib can block the PKCα/ZFP64/CSF1 axis to reset the tumor microenvironment and restore sensitivity to anti-PD-1.^[30]^ However, studies have shown that drug resistance and adverse effects of lenvatinib greatly hinder the efficacy of clinical treatment of HCC,^[31]^ and the use of targeted agents alone not only fails to achieve the desired effect, but also the adverse effects brought about by it are an additional burden for patients, so combination therapy is a trend in clinical treatment of HCC,^[32]^ to improve the response rate of tumors to treatment, prolong PFS and OS, and specific combination therapies need to be evaluated for effectiveness and safety through clinical trials.

## 5. Conclusion

In this study, we evaluated the information of literature from different years, countries, institutions, authors, disciplines, and journals by detailed bibliometric analysis of TKIs and HCC and analyzed the topic development and future research hotspots. Our study found that the field has been receiving attention since 2004. Our study provides basic information on research in the field and identifies potential partners for interested researchers. The current research hotspots mainly include the study of inflammatory mechanisms, immune mechanisms, related diseases, and related cytokines. The application of TKIs for liver cancer treatment and TK combination with multiple therapeutic regimens are at the forefront of research in this area and are emerging.

## Author contributions

**Data curation:** Hejun Mao.

**Writing – original draft:** Wurihan Wu.

**Resources:** Jian Song.

**Software:** Jian Song.

**Funding acquisition:** Fan Yang.

**Supervision:** Fan Yang.

**Visualization:** Fan Yang.
